# The role of prevertebral soft tissue swelling in dysphagia after anterior cervical corpectomy fusion: change trends and risk factors

**DOI:** 10.1186/s12891-023-06843-7

**Published:** 2023-09-09

**Authors:** Yanyan Ma, Peiming Sang, Binhui Chen, Jie Li, Dikai Bei

**Affiliations:** 1 Department of Gastroenterology, Lihuili Hospital of Ningbo Medical Center, Ningbo, Zhejiang PR China; 2 Department of Orthopedic Surgery, Lihuili Hospital of Ningbo Medical Center, #57, Xingning Road, Yinzhou District, Ningbo, Zhejiang PR China

**Keywords:** Dysphagia, Prevertebral soft tissue swelling (PSTS), Anterior cervical corpectomy fusion (ACCF), Cervical spine surgery, Risk factor, Change trend

## Abstract

**Objectives:**

This study aimed to analyze the change trends of prevertebral soft tissue swelling (PSTS) for anterior cervical corpectomy fusion(ACCF) and to evaluate the risk factors of PSTS for postoperative dysphagia.

**Methods:**

There were 309 patients with degenerative cervical diseases who were treated with ACCF from November 2015 and September 2019 in our hospital. According to the symptom of swallowing function after ACCF, those were divided into the dysphagia group and the normal-swallowing function group. Cervical computed tomography(CT) was analyzed, and radiological evaluation of the prevertebral soft tissue was measured between the antero-inferior corner of each vertebral body and the air shadow of the airway through CT mid-sagittal slice images before operation and after operation(one week, one month, eight months and twelve months).

**Results:**

The incidence of dysphagia after ACCF was 41.1%. 120 of 127(94.5%) patients had dysphagia disappeared at the 8 months after ACCF, and all disappeared at the 12 months. In both groups, PSTS would be biggest at 1 week postoperatively comparing to the preoperative, and then get smaller from 1 week to 12 months postoperatively (*p* < 0.05). After 12 months of operation, the PSTS of all cervical spinal levels would get equal to the preoperative size in the normal-swallowing function group, while the PSTS in dysphagia group would get equal only in C5-7 levels. The PSTS of preoperative C6 level and postoperative C2 level were more closely related to the present of postoperative dysphagia (OR: 9.403, 95%CI: 2.344–37.719, OR: 3.187, 95%CI: 1.78-5.705). It was more important to predict postoperative dysphagia using the value of PSTS at preoperative C6 level and postoperative C2 level, with the cutoff threshold for the PSTS of preoperative C6 level ≦1.51 cm and postoperative C2 level ≦1.3915 cm, which could get sensitivity & specificity 66.929% and 61.54%, 77.17% and 64.29%, respectively.

**Conclusion:**

Our study showed that the increasing of the PSTS after ACCF should be considered as a risk factor of dysphagia after surgery. With the recovery of PSTS over time, the incidence of postoperative dysphagia decreases. The PSTS of preoperative C6 level and and postoperative C2 level should play an important part in predicting the risk of postoperative dysphagia.

## Introduction

Currently, anterior cervical corpectomy fusion(ACCF) has been a common surgery. However, the dysphagia after surgery is one of the common complications and has been concerned in the recent years [1]. Although the incidence of dysphagia after anterior cervical surgery was underestimated by spine surgeons [2], some studies have reported the incidence of dysphagia after surgery between 47-60% [2–7]. And severe dysphagia in the early postoperative time has been reported in 7% of patients [4, 8].

Some studies have shown that soft-tissue swelling is considered as a cause of dysphagia after anterior cervical surgery [6, 8, 9]. However, that have evaluated the PSTS postoperatively, which were not accurate according to cervical plain lateral radiography. In order to access PSTS precisely, this study would evaluate the change trends of the PSTS through CT mid-sagittal slice images, and assess the period that the PSTS would last. It also evaluate the risk factors of the PSTS in the postoperative dysphagia, and analyze the relationship between the PSTS and dysphagia.

## Materials

Inclusion criteria: ① cervical degenerative disease should be operated at two adjacent levels, ② anterior lesions that compress spinal cord or spinal nerve should be removed with the surgery, ③ the lesions could not be treated by intervertebral approach, or the intervertebral space was very narrow, ④ patient’s symptoms and signs were consistent with medical imaging, ⑤ patients without preoperative dysphagia, ⑥ patients were followed up for one year after operation, ⑦ one level of ACCF. Exclusion criteria: ① cervical revision surgery, ② cervical infection or tumor, ③ esophageal perforation, ④ the failure of internal fixation after surgery, ⑤ other surgeries except one level of ACCF.

There were 309 patients with degenerative cervical diseases who have been undergone with ACCF between November 2015 and September 2019 at our hospital. The study protocol has been approved by the Hospital’s Research Ethics Board (Number: 2017025, Date: December, 23rd, 2017).

## Method

The surgery of anterior cervical corpectomy fusion was achieved under general anesthesia and approached from the right side in all patients. A Smith-Robinson incision was made on the right side of the neck, the right platysma was transected and then dissected along the dorsal surface of the platysma. The muscle flap was separated along the inner edge of the right sternocleidomastoid muscle. The carotid artery was retracted laterally, the esophagus and trachea were pulled medially. The prevertebral fascia was incised carefully. The exposure was used with two retractors beneath the longus colli muscles on the both sides by the fluoroscopic guidance into the target cervical vertebrae. A segmental vertebral, two adjacent disc materials, endplate cartilage and posterior longitudinal ligament were removed to decompress nerve completely. Appropriate titanium mesh (Stryker Spine S.A.S, VBOSS, France) full of autologous cancellous bone was inserted into the intervertebral space and a plate (Aesculap, ABC, Germany) or (MedioxOrvosiMuszergyartoKft, Anterior Spinal Plate, Hungary)was fixed. Closed suction drain was used and the drain was removed two days after operation.

We evaluated the swallowing quality at one week, one month, eight months and twelve months after ACCF according to the standard of the Swallowing-Quality of Life(SWAL-QOL) [1, 10]. The SWAL-QOL questionnaire included the categories regarding the impact of dysphagia from the patient’s perspective: burden, eating duration, eating desire, symptom frequency, food selection, communication, fear, mental health, social, fatigue, and sleep. The frequence of symptoms were divided into almost always, often, sometimes, hardly ever, or never. Whether the postoperative dysphagia was happened or not according to the result of the patients’ feeling and the frequence of symptoms.

**Fig. 1:**
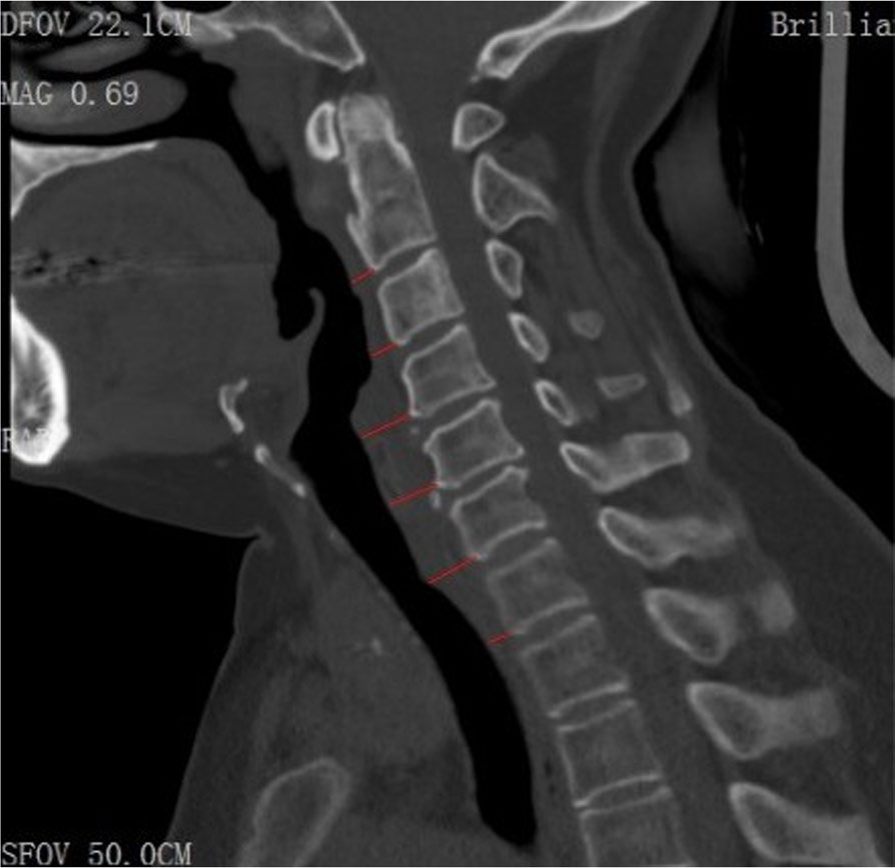
The PSTS was measured between the antero-inferior corner of each vertebral body and the air shadow of the airway through CT mid-sagittal slice images

The brief complaint, physical examination and radiological exams, contained X-ray, CT and MRI, were tested preoperatively. After the operation, Cervical spinal column CT and X-ray were performed at one week, one month, eight months and twelve months. Radiological evaluation of the prevertebral soft tissue was measured between the antero-inferior corner of each vertebral body and the air shadow of the airway through CT mid-sagittal slice images (Fig. 1).

### Statistical analysis

Statistical analysis was performed with the SPSS ver.16.0 (SPSS Inc., Chicage, IL, USA). The analysis of variance for repeated measurement data and LSD test were used to determine significant differences of prevertebral soft tissue swelling at the different periods. Chi-quared test was used to evaluate significant differences among surgical levels. *P* < 0.05 indicated significant differences. Logistic regression models were created to assess the risk factors of prevertebral soft tissue swelling perioperatively in dysphagia after ACCF. Receiver operating characteristic (ROC) curves were used to determine optimal threshold values of prevertebral soft tissue swelling for dysphagia after ACCF.

## Results

There were 309 patients divided into dysphagia group and normal-swallowing function group (Table 1). There were no significant differences about gender, age and surgical levels between the two groups (*P* > 0.05).

**Table 1:** Patients’ basic characteristics

	Dysphagia group	Normal-swallowing function group	*p*
Male	79	131	
Female	48	51	0.0829
Mean age	59.299 ± 10.227	59.8352 ± 8.247	0.6113
Surgical levels			0.9679
C3	6	7	
C4	30	45	
C5	38	55	
C6	40	60	
C7	13	15	

The incidence of dysphagia after ACCF was 41.1%. 120 of 127(94.5%) patients had dysphagia disappeared at the 8 months after ACCF, and all disappeared at the 12 months. The PSTS measurement in dysphagia group after ACCF was shown at Table 2.

**Table 2:** The PSTS measurement (cm) for dysphagia group after ACCF at different periods (pre-operation, 1 week, 1 month, 8 months, 12 months after operation)

	Pre-operation	1 week after operation	1 month after operation	8 months after operation	12 months after operation
C2	0.521 ± 0.248	1.972 ± 1.221^a^	1.176 ± 0.548^b^	0.9006 ± 0.359^c^	0.775 ± 0.291^e^
C3	0.509 ± 0.242	1.999 ± 1.106^a^	1.106 ± 0.567^b^	0.972 ± 0.49^g^	0.794 ± 0.347^d,e^
C4	1.094 ± 0.706	2.65 ± 1.685^a^	1.792 ± 1.096^b^	1.491 ± 0.727^c^	1.387 ± 0.688^e^
C5	1.471 ± 0.384	2.527 ± 1.465^a^	1.922 ± 0.78^b^	1.717 ± 0.496^c^	1.649 ± 0.446^f^
C6	1.628 ± 0.458	2.536 ± 1.417^a^	1.991 ± 0.772^b^	1.847 ± 0.601^g^	1.789 ± 0.564^f^
C7	1.422 ± 0.512	2.383 ± 1.312^a^	1.831 ± 0.779^b^	1.644 ± 0.549^g^	1.565 ± 0.522^f^

^a^*p* < 0.01 vs pre

^b^ < 0.01 vs 1 week

^c^ < 0.05 vs 1 month

^d^ < 0.05 vs 8 months

^e^ < 0.05 vs pre

^f^ > 0.05 vs pre

^g^ > 0.05 vs 1 month

Due to the repeated measurement data of the PSTS, the Mauchly’s test of sphericity for dysphagia group was measured, *P* < 0.05. Multivariate analysis of variance with the method of Greenhouse-Geisser modified test was shown at Table 3. At the same cervical spinal level, the comparison between each other was described at Table 1 for different periods.

**Table 3:** Multivariate analysis of variance for dysphagia group

Source	Type III sum of squares	df	Mean Square	F	Sig
Period	683.96	1.361	502.394	501.362	2.756 × 10^–115^
Level	495.596	5	99.119	52.2	1.506 × 10–46
Period*level	31.617	6.807	4.645	4.635	4.94 × 10–5
Error(period)	1031.339	1029.22	1.002		
Error(level)	1435.523	756	1.899		

*df* degrees of freedom, *sig* significance

The PSTS for dysphagia group was biggest at 1 week postoperatively and then would get smaller from 1 week to 8 months postoperatively (*p*<0.05). In the terms of change for C5, C6, C7 level, the PSTS for 12 months after operation was equal with the preoperative. But it would not been recovered to the preoperative at the C2, C3, C4 level for 12 months after operation.

In the normal-swallowing function group after ACCF, The PSTS measurement was shown at Table 4.

**Table 4:** The PSTS measurement (cm) of different levels for normal-swallowing function group at different periods (pre-operation, 1 week, 1 month, 8 months, 12 months after operation

	Pre-operation	1 week after operation	1 month after operation	8 months after operation	12 months after operation
C2	0.378 ± 0.1605	1.412 ± 0.736^a^	0.92 ± 0.4194^b^	0.654 ± 0.252^c,e^	0.3888 ± 0.15^d,h^
C3	0.396 ± 0.1566	1.648 ± 0.859^a^	1.033 ± 0.4407^b^	0.6712 ± 0.189^c,e^	0.41 ± 0.156^d,h^
C4	0.79895 ± 0.483	2.062 ± 0.9319^a^	1.698 ± 1.2016^b^	1.118 ± 0.416^c,e^	0.814 ± 0.425^d,h^
C5	1.2825 ± 0.497	2.2234 ± 1.2958^a^	2.104 ± 1.726^b^	1.479 ± 0.473^c,f^	1.28 ± 0.496^g,h^
C6	1.3459 ± 0.48	2.2346 ± 1.178^a^	2.125 ± 1.426^b^	1.5522 ± 0.4564^c,e^	1.343 ± 0.477^d,h^
C7	1.272 ± 0.646	2.0367 ± 1.2648^a^	1.6796 ± 0.7704^b^	1.403 ± 0.6586^c,f^	1.2529 ± 0.6446^g,h^

^a^*p* < 0.01 vs pre

^b^ < 0.01 vs 1 week

^c^ < 0.05 vs 1 month

^d^ < 0.05 vs 8 months

^e^ < 0.05 vs pre

^f^ > 0.05 vs pre

^g^ > 0.05 vs 8 months

^h^ > 0.05 vs pre

Due to the repeated measurement data of the PSTS, the Mauchly’s test of sphericity for the normal-swallowing function group was measured, *P*<0.05. Multivariate analysis of variance with the method of Greenhouse-Geisser modified test was shown at Table 5.

**Table 5:** Multivariate analysis of variance for the normal-swallowing function group

Source	Type III sum of squares	df	Mean Square	F	*P*
Period	887.989	1.942	457.319	548.223	6.152 × 10^–188^
Level	809.76	5	161.952	122.206	1.124 × 10^–102^
Period*level	43.306	9.7086	4.4606	5.347	1.1435 × 10^–7^
Error(period)	1759.06	2108.718	0.834		
Error(level)	1439.207	1086	1.3252		

*df* degrees of freedom, *sig* significance

The PSTS for the normal-swallowing function group was biggest at 1 week postoperatively and then got smaller from 1 week to 8 months (*p*<0.05). The PSTS at 8 months was equal with that of pre-operation at C5, C7 level. The PSTS at 12 months was recovered to the preoperative size at C2, C3, C4, C6 level. The results were shown at Table 4.

In order to assess the risk factors about the dysphagia after ACCF using logistic regression model, it was important to evaluate the PSTS at preoperation, 1 week after operation. The relationship between the PSTS and the incidence of postoperative dysphagia was analyzed and the result was shown at Table 6. The incidence of postoperative dysphagia was related with the PSTS at preoperative C4, C6 level and postoperative C2, C5, C7 level.

**Table 6:** Logistic regression for PSTS

	B	S.E,	Wald	df	sig	OR	95% CI of OR	
							Lower	Upper
Preoperative C2 level	1.85	1.274	2.107	1	0.147	6.359	0.523	77.279
Preoperative C3 level	-0.399	1.233	0.105	1	0.746	0.671	0.06	7.52
Preoperative C4 level	0.686	0.336	4.174	1	0.041	1.986	1.028	3.838
Preoperative C5 level	-0.592	0.699	0.784	1	0.376	0.553	0.149	2.051
Preoperative C6 level	2.241	0.709	9.998	1	0.002	9.403	2.344	37.719
Preoperative C7 level	-1.018	0.546	3.472	1	0.062	0.361	0.124	1.054
Postoperative C2 level	1.159	0.297	15.211	1	9.614 × 10^–5^	3.187	1.78	5.705
Postoperative C3 level	-0.215	0.327	0.432	1	0.511	0.807	0.425	1.53
Postoperative C4 level	0.221	0.263	0.701	1	0.402	1.247	0.744	2.089
Postoperative C5 level	-1.017	0.489	4.332	1	0.037	0.362	0.139	0.942
Postoperative C6 level	-0.59	0.513	1.321	1	0.25	0.555	0.203	1.516
Postoperative C7 level	1.059	0.465	5.178	1	0.023	2.883	1.158	7.175
Constant	-3.399	0.608	31.25	1	2.269 × 10^–8^	0.033		

*B* Beta, *S.E* Standard Error, *df* Degrees of freedom, *sig* significance, *OR* Odds Ratio, *CI* Confidence interval

**Fig. 2:**
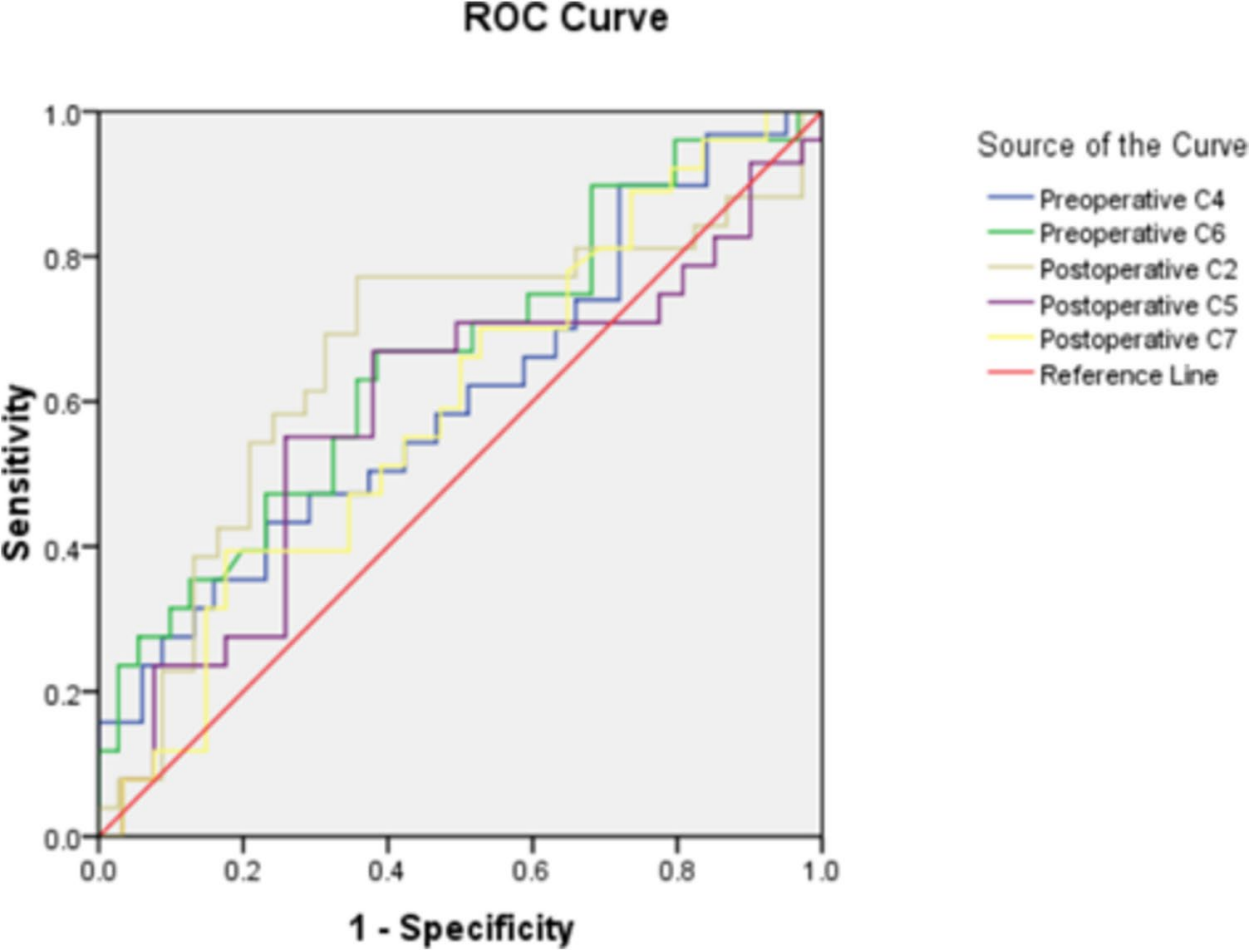
ROC analysis of the PSTS. ROC: receiver operating characteristic

ROC analysis of the PSTS of preoperative C4, C6 level and postoperative C2, C5, C7 level was tested (Fig. 2). The cut of value of the PSTS of the above level was showed at Table 7. It was more important for predicting postoperative dysphagia using the value of PSTS at preoperative C6 level and postoperative C2 level, with the cutoff threshold for the PSTS of preoperative C6 level ≦1.51cm and postoperative C2 level ≦1.3915cm, which could get sensitivity & specificity 66.929% and 61.54%, 77.17% and 64.29%, respectively.

**Table 7:** ROC analysis of cut off value for the PSTS

Period	Area	*P* value	Cut off value	Sensitivity	Specificity
Preoperative C4 level	0.6069	0.001385	1.222	43.3%	76.92%
Preoperative C6 level	0.6576	2.416 × 10^–6^	1.51	66.929%	61.54%
Postoperative C2 level	0.6616	1.341 × 10^–6^	1.3915	77.17%	64.29%
Postoperative C5 level	0.5849	0.0111	2.395	55.12%	74.18%
Postoperative C7 level	0.593	0.00542	2.5125	39.37%	82.42%

## Discussion

Dysphagia is a common complication after anterior cervical spinal surgery. In our study, the incidence of postoperative dysphagia was 41.1% in ACCF, which was similar to the present study. Previous studies have reported the incidence of dysphagia after general anterior cervical surgery between 47-60% [2–7]. And severe dysphagia in the early postoperative time has been reported in 7% of general anterior cervical patients [4, 8], which could affect the quanlity of patients’ life heavily. So the doctors should pay more attention to the dysphagia after anterior cervical spinal surgery.

Previous studies have described the role of prevertebral soft tissue after anterior cervical spinal surgery [11–14].And Miles et al. [15] have revealed that the PSTS size of larger than half of the diameter of the vertebral body in the retropharyngeal space should be abnormal.

The prevertebral soft tissue swelling after anterior cervical spine surgery was associated with postoperative dysphagia. Some studies [16–20] showed that an increase in prevertebral soft tissue may cause dysphagia after anterior cervical spine surgery, and positive C3 or C4 PSTS at 2 or 3 days after surgery were more likely to have postoperative dysphagia. To decrease the tissue injury, some studies [21, 22] suggested intermittent release of retractors should be used in order to minimize the injury of compression-associated tissue ischemia.

In our study, the change of PSTS at lower cervical levels (C5, C6, C7) was different from that at upper cervical levels (C2, C3, C4). In both group, the PSTS at lower cervical levels recovered more quickly than that at upper cervical levels, and the PSTS for upper cervical levels in dysphagia group could not return to normal. The reasons for these changes were resulted from the differences of anatomy. Kyung-Jin Song, et al. [19] also found that the lower vertebral levels experienced little change postoperatively when compared with the upper cervical spine. This is most likely because of the more constrained anatomy of the lower cervical spine.

It was very difficult to assess the degree of post-dysphagia by some objectively medical examinations. Leonard Haller et al. [23] thought that there was inadequate screening and otolaryngology follow-up for patients with Post-operative dysphagia in anterior cervical discectomy and fusion.

In general, the dysphagia was temporary and returned to normal in 12 months with the recovery of PSTS through our study, which is the normal trend. In case of the dysphagia was getting worse or has been for a long time, it was very important to analyze the reason, in order to avoid the occurrence of esophageal perforation that could result in the deadth with a higher rate.

Some studies [24, 25] showed that surgery involving C3-4, multilevel-segments surgery(≥ 3 segments), long operative times, female sex, older age, use of an anterior cervical plate, and revision surgery were all significant risk factors for the development of dysphagia after cervical spine surgery.

In our study, it was more significant to predict the risk of postoperative dysphagia using the value of PSTS at preoperative C6 level and postoperative C2 level, with the cutoff threshold for the PSTS at preoperative C6 level ≦ 1.51cm and postoperative C2 level ≦ 1.3915cm, which could get sensitivity & specificity 66.929% and 61.54%, 77.17% and 64.29%, respectively. So prior to the surgery, it was very important to predict the risk of postoperative dysphagia according the value of preoperative PSTS at C6 level. And during operation, it was necessary to evaluate the PSTS at C2 level using the C arm X-ray machine perspective in order to estimate the incidence of postoperative dysphagia.

The PSTS at preoperative C6 level and postoperative C2 level were related to the occurrence of dysphasia after ACCF, there were some possible reasons. First, at C6 level, there was the beginning of esophagus which was narrow congenitally. Second, at C2 level, the PSTS was injured during trachea cannula. Third, at C2 level, the position of PSTS was experienced more change due to the pulling during surgery, compared to the lower ACCF, because of the more constrained anatomy of the lower cervical spine.

If the risk of postoperative dysphagia was higher according to the preoperative value of PSTS at preoperative C6 level, some preventive actions would be taken. Some studiesl [25, 26] suggested that adequately preoperative tracheal traction, smoking cessation, endotracheal tube cuff pressure of 20 mmHg during sugery, avoiding routine use of rhBMP-2, use of a zero-profile implant, use of Zephir plate, use of a new cervical retractor, intraoperative steroid administration, use of disc arthroplasty, avoidance of prolonged operating time, avoidance of over-enlargement of cervical lordosis, and decreasing the surgical segments.

### Limitations

This study has some significant limitations. First, some factors are not considered, such as the degrees of traction during surgery, the difficulty of each surgery, the thickness of the plate used, the short follow-up period, the surgical levels. Second, the cutoff point of PSTS is not the best as the sensitivity & specificity are lower.

## Conclusions

In conclusion, dysphagia after anterior cervical corpectomy fusion is a common complication and multifactorial. The PSTS after surgery should be considered as a risk factor of dysphagia after surgery. And with the recovery of PSTS, the incidence of postoperative dysphagia decreases. The value of PSTS at preoperative C6 level and postoperative C2 level are more important for predicting postoperative dysphagia.

## Data Availability

The data generated or analyzed during this study are contained in this published article.

## Abbreviations

PSTS: Prevertebral soft tissue swelling
ACCF: Anterior cervical corpectomy fusion
SWAL-QOL: Swallowing-Quality of Life
ROC: Receiver operating characteristic
CT: Computed tomography
